# Data on morphological features of mycosis induced by *Colletotrichum nymphaeae* and *Lecanicillium longisporum* on citrus orthezia scale

**DOI:** 10.1016/j.dib.2016.05.008

**Published:** 2016-05-12

**Authors:** Gabriel Moura Mascarin, Juan Humberto Guarín-Molina, Steven Paul Arthurs, Richard Alan Humber, Rafael de Andrade Moral, Clarice Garcia Borges Demétrio, Ítalo Delalibera

**Affiliations:** a Embrapa Arroz e Feijão, Rodovia GO-462, Km 12, Zona Rural, C.P. 179, 75375-000 Santo Antônio de Goiás, GO, Brazil; b Corporación Colombiana de Investigación Agropecuária (CORPOICA), Centro de Investigación “La Selva”, A.A. 100 Rionegro, Antioquia, Colombia; c University of Florida/IFAS, Mid Florida Research and Education Center, Apopka, FL 32703, USA; d United States Department of Agriculture, Agricultural Research Service, Emerging Pests and Pathogens Research, Robert W. Holley Center for Agriculture & Health, 538 Tower Road, Ithaca, NY 14853, USA; e Department of Exact Sciences, University of São Paulo, Av. Pádua Dias 11, CP 9, 13418-900 Piracicaba, SP, Brazil; f Department of Entomology and Acarology, University of São Paulo, Av. Pádua Dias 11, CP 9, 13418-900 Piracicaba, SP, Brazil

**Keywords:** Insect mycosis, Conidia, Conidiophores, Microscopy, Biological control, Ascomycetous fungi

## Abstract

We describe symptoms of mycosis induced by two native fungal entomopathogens of the citrus orthezia scale, *Praelongorthezia praelonga* (Hemiptera: Ortheziidae), an important pest of citrus orchards. The data presented in this article are related to the article entitled “Seasonal prevalence of the insect pathogenic fungus *Colletotrichum nymphaeae* in Brazilian citrus groves under different chemical pesticide regimes” [1]. The endemic fungal pathogen, *C. nymphaeae*, emerges through the thin cuticular intersegmental regions of the citrus orthezia scale body revealing orange salmon-pigmented conidiophores bearing conidial masses, as well as producing rhizoid-like hyphae that extend over the citrus leaf. By contrast, nymphs or adult females of this scale insect infected with *Lecanicillium longisporum* exhibit profuse outgrowth of bright white-pigmented conidiophores with clusters of conidia emerging from the insect intersegmental membranes, and mycosed cadavers are commonly observed attached to the leaf surface by hyphal extensions. These morphological differences are important features to discriminate these fungal entomopathogens in citrus orthezia scales.

**Specifications Table**

**Table:** 

Subject area	Biology
More specific subject area	Biological control
Type of data	Image (microscopy)
How data was acquired	Stereo-microscope, phase-contrast microscope, scanning electron microscope, in vivo
Data format	Raw
Experimental factors	Field collected insects infected with *Lecanicillium longisporum* or *Colletotrichum nymphaeae* were photographed using a stereo-microscope at 10 to 30× magnification, while their conidia were prepared for phase-contrast or scanning electron microscopic examinations.
Experimental features	Photographs and microphotographs portraying typical signs of mycosis of two fungal pathogens infecting the citrus orthezia scale
Data source location	n/a
Data accessibility	Data are provided with this article

## Value of the data

• Data show symptoms of two important fungal diseases in the citrus orthezia scale caused by *C. nymphaeae* and *L. longisporum*.
• Simple and practical recognition of these entomopathogenic fungi can be achieved on the basis of their conspicuous morphological characters, including conidiophores and conidia, from mycosed scale insects.
• The importance of using morphological features based on symptoms of fungal diseases can serve as a guideline to facilitate recognition and quantification of these pathogens in citrus orthezia scale populations.

## 1 Data

The data display photos illustrating typical morphological features (conidiophores, conidia and rhizoid-like hyphae) of infected citrus orthezia scales with two native fungal pathogens, namely *C. nymphaeae* and *L. longisporum* (Fig. S1). Images were acquired by using dissecting stereo-microscope, light microscope and scanning electron microscope.

## 2 Experimental design, materials and methods

Field collected insects infected with *L. longisporum* or *C. nymphaeae* from citrus groves located in a commercial farm (Matão, SP, Brazil) were taken to the laboratory to record the morphological features of mycosis in individuals of the citrus orthezia scale. Images of mycosed insects were acquired by using a stereo-microscope at 10–30× magnification, while images of conidiospores were taken by phase-contrast and scanning electron microscopy. These morphological characters related to signs of fungal diseases were used as a guideline for identification and quantification of these fungal entomopathogens in this insect population during field surveys [1].

## Footnotes

Appendix A Supplementary data associated with this article can be found in the online version at http://dx.doi.org/10.1016/j.dib.2016.05.008.
