# Supplementary material for: Data on morphological features of mycosis induced by Colletotrichum nymphaeae and Lecanicillium longisporum on citrus orthezia scale
Source: Data Brief. 2016 May 12;8:49–51. doi: 10.1016/j.dib.2016.05.008 (PMC4885018; doi:10.1016/j.dib.2016.05.008)
Supplement: Supplementary material [file mmc1.doc]

St. Antonio de Goiás, GO, Brazil. March 8th, 2016

**Declaration of interest**

We have no conflict of interest to declare.
