# Supplementary material for: Data on morphological features of mycosis induced by Colletotrichum nymphaeae and Lecanicillium longisporum on citrus orthezia scale
Source: Data Brief. 2016 May 12;8:49–51. doi: 10.1016/j.dib.2016.05.008 (PMC4885018; doi:10.1016/j.dib.2016.05.008)
Supplement: Supplementary material [file mmc2.pdf]

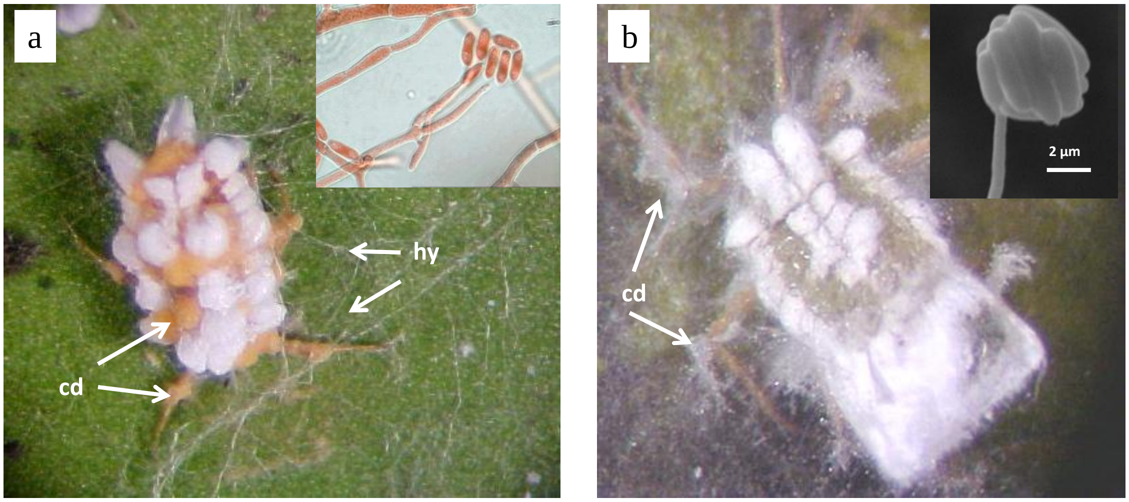

Figure S1. Signs of mycoses on the citrus scale *Praelongorthezia praelonga*. a) Nymph killed by *Colletotrichum nymphaeae s*howing typical orange salmon-pigmented conidial masses (cd) emerging between insect's segments, and rhizoid-like hyphal extensions (hy) extending onto the citrus leaf; b) Nymph infected with *Lecanicillium longisporum* showing profuse external growth and bright white-pigmented conidiophores bearing clusters of conidia (cd) emerging from the insect's leg joints. Top-right images portray typical conidia cluster formed by conidiogenous cells.
